# Low-Coverage Whole-Genome Resequencing in Livestock and Poultry: Statistical Foundations, Applications and Future Directions

**DOI:** 10.3390/biology15161370

**Published:** 2026-08-12

**Authors:** Jianqing Zhao, Tuersunayi Muhetaer, SimubatiGuli Shahatinuer, JingesiKailede Nuerlan, Mina Nuertai, Wuxixiaer Kanixi, Wei Wang, Junde Ma

**Affiliations:** 1 College of Animal Science, Xinjiang Agricultural University, Urumqi 830052, China; zhao2021@nwafu.edu.cn (J.Z.); 16699785168@163.com (T.M.); 2 Xinjiang Gongnaisi Sheep Farm Co., Ltd., Yining 835808, China; 15276608231@163.com (S.S.); 18290973646@163.com (J.N.); 13669924475@163.com (M.N.); 18195893927@163.com (W.K.); 3 Shaanxi Key Laboratory of Molecular Biology for Agriculture, College of Animal Science and Technology, Northwest A&F University, Yangling 712100, China; wangweixn007@nwafu.edu.cn

**Keywords:** low-coverage whole-genome sequencing, livestock genomics, genotype imputation, genomic selection, population genomics

## Abstract

Low-coverage whole-genome resequencing (lcWGS) is emerging as a powerful population-scale genomic strategy for livestock and poultry research. By integrating sparse sequencing reads with genotype likelihoods, haplotype information and imputation models, lcWGS enables genome-wide variant discovery and genetic inference across large animal cohorts. This feature is particularly valuable for breeding populations, indigenous breeds and conservation resources, where broad sampling is essential for capturing population-specific variation and linking genomic diversity with economically and adaptively important traits. In this review, we synthesize the statistical foundations, analytical workflows and major applications of lcWGS in livestock and poultry genomics. We discuss how lcWGS supports genetic diversity assessment, population structure analysis, genome-wide association studies, genomic selection, selection-signature detection, environmental adaptation research and genetic resource conservation. We further highlight the importance of coordinated study design, including sequencing depth, sample size, reference-panel construction, imputation strategy, phenotype quality and downstream analytical models. Beyond its role as a cost-efficient genotyping approach, lcWGS provides a flexible framework for integrating population genomics with functional annotation, multi-omics resources, long-read assemblies, graph pan-genomes and interpretable prediction models. These developments are expanding the potential of lcWGS from variant discovery toward biological interpretation, precision breeding, climate-resilient animal production and the sustainable management of livestock and poultry genetic resources.

## Simple Summary

Low-coverage whole-genome resequencing (lcWGS) provides a cost-effective approach for obtaining genome-wide information from large livestock and poultry populations. By combining low-depth sequencing data with genotype likelihoods, haplotype information and imputation methods, lcWGS enables studies of genetic diversity, population structure, trait-associated variants, genomic selection and conservation of animal genetic resources. This review summarizes the statistical principles, applications and future opportunities of lcWGS in animal genomics, emphasizing study design, quality control and integration with functional genomic resources.

## 1. Introduction

Whole-genome resequencing has become a central tool for connecting genetic variation with traits of agricultural and evolutionary importance. However, high-depth whole-genome sequencing remains expensive when applied to the sample sizes required for modern animal breeding, conservation genetics and population genomics. Low-coverage whole-genome resequencing (lcWGS), typically conducted at approximately 0.1× to 5× depth per individual, addresses this constraint by distributing sequencing effort across many samples and recovering genotypes through genotype likelihoods, haplotype sharing and imputation [1,2,3,4,5]. This design is especially attractive in livestock and poultry because large cohorts are often available through breeding programs, experimental crosses, conservation herds, or gene banks, whereas high-quality, species- or breed-specific SNP arrays are not always available.

The conceptual advantage of lcWGS is that it converts a sequencing problem into a statistical inference problem. Instead of demanding complete genotype information for every animal, lcWGS estimates genotype probabilities from sparse read data and leverages linkage disequilibrium, pedigree relatedness and reference haplotypes to infer missing or uncertain genotypes [4,6,7,8]. As a result, lcWGS can provide denser and less ascertainment-biased marker sets than commercial SNP arrays, while enabling substantially larger sample sizes than high-depth sequencing for the same budget [2,6]. This trade-off can increase power for population-level analyses, association mapping and genomic prediction, particularly when causal variants are poorly tagged by array markers or when indigenous breeds carry population-specific alleles [9,10].

The approach also has important boundaries. Low read depth reduces confidence in heterozygote detection, creates extensive missingness and limits the reliable discovery of rare variants, copy-number variants and complex structural variants [11,12]. Analytical pipelines must therefore avoid treating lcWGS data as if they were high-depth genotypes. Genotype likelihood-based methods, probabilistic variant calling and imputation-aware downstream analyses are essential for minimizing bias in allele-frequency estimation, GWAS, selection scans and genomic relationship estimation [11,12]. In addition, imputation accuracy can decline when reference panels are small [13,14,15], genetically distant from the target population or not representative of local breeds and poultry lines [9,10].

In this review, we examine lcWGS as an integrated experimental and computational strategy for livestock and poultry genomics. Unlike general discussions of lcWGS [1], we focus on the biological and breeding contexts of farm animals, where long-range linkage disequilibrium, family structure, breed-specific haplotypes, reference-population design [9,13], indigenous genetic resources and commercial data restrictions shape both study design and downstream interpretation. We first summarize the statistical principles, sequencing-depth considerations, cost-depth trade-offs and quality-control requirements of lcWGS. We then synthesize its major applications in animal breeding, evolutionary genomics and conservation, with emphasis on how different analytical choices affect biological inference. Finally, we discuss emerging opportunities for integrating lcWGS with functional annotation, long-read sequencing, pan-genome resources and interpretable prediction models to support trait discovery, precision breeding and sustainable management of animal genetic resources.

## 2. Methodological Framework for lcWGS

A rigorous lcWGS study begins before sequencing. The key design question is not simply how low the sequencing depth can be, but how the available budget should be allocated among depth, sample size, reference-panel construction, phenotyping and computational validation. We therefore distinguish four practical depth ranges. Ultra-low-pass sequencing (approximately 0.1–0.5×) is most appropriate when the aim is inexpensive common-variant genotyping in very large cohorts with strong reference support. Low-pass sequencing (approximately 0.5–1.5×) is often a useful compromise for GWAS and genomic prediction when imputation accuracy can be validated. Intermediate low coverage (approximately 1.5–4×) improves within-cohort variant discovery and low-frequency variant tagging but increases cost. Coverage of approximately 4–5× approaches medium coverage and should be justified by specific needs such as weaker reference panels, mixed-breed cohorts or limited validation data.

A simple budget expression makes the trade-off explicit: total sequencing cost is approximately proportional to N × d × G, where N is the number of animals, d is the intended depth and G is the mappable genome size, with additional library, labor, storage and analysis costs. If the same budget is used, reducing depth from 4× to 1× can in principle permit about four times as many animals to be sequenced, but only if imputation quality, phenotype quality and reference-panel match remain adequate. Because vendor prices vary by country, platform, sample number and genome size, the most transparent way to report cost-effectiveness is to provide both actual local costs and relative cost units. For example, taking a routine medium-density SNP array as one cost unit, 0.5–1× lcWGS may be similar to or modestly higher than array genotyping in large batches, 4–5× lcWGS is usually several cost units, and 30× WGS is generally an order of magnitude higher. This relative framing prevents overgeneralization while still allowing readers to evaluate the depth-sample-size trade-off (Table 1).

Experimental design should account for DNA quality, library complexity, read length, multiplexing level, duplicate rate, insert-size distribution and the expected genetic relationship among samples. In livestock, close pedigree relationships can improve imputation through shared haplotypes, whereas highly admixed or geographically structured populations may require broader reference panels and careful stratified validation. In poultry, short generation intervals, strong selection and line-specific haplotypes can create long-range linkage disequilibrium that is beneficial for imputation within lines but may reduce transferability across commercial and indigenous populations.

**Figure 1:**
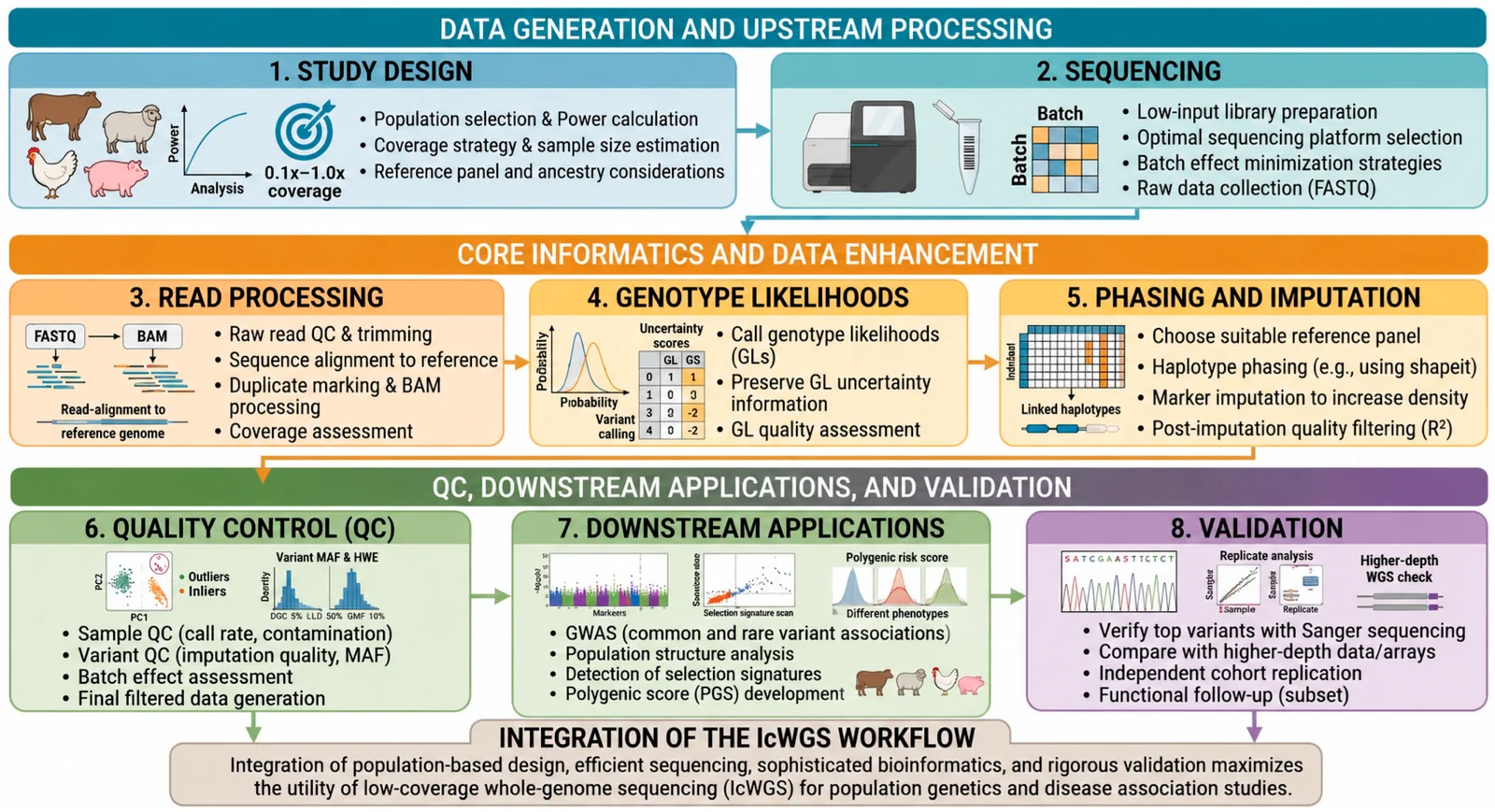
End-to-end lcWGS workflow. The workflow emphasizes study-design decisions, low-pass sequencing, uncertainty-aware read processing, genotype-likelihood estimation, phasing and imputation, multi-layer QC, downstream analyses and validation.

The bioinformatics workflow usually includes read quality control, trimming when necessary, alignment to a reference genome, duplicate marking, base-quality recalibration or equivalent filtering, genotype-likelihood estimation, variant discovery, phasing, imputation and post-imputation quality control. GATK, SAMtools/BCFtools and ANGSD remain widely used for read processing, genotype likelihoods and low-depth population-genetic analyses [11,16,17]. For imputation, GLIMPSE/GLIMPSE2 improves sparse conditioning and scalability with large reference panels [3,4], QUILT is read-aware and reference-panel based [18], Beagle 5 is widely used for phased reference-panel imputation [19], STITCH can be useful when external reference panels are weak [5], and BaseVar-STITCH has been applied in livestock lcWGS studies [9,10,20]. For genotype-likelihood-based population genetics, ANGSD-derived workflows can be paired with PCAngsd [21], NGSadmix [22], ngsRelate [23] and realSFS/winsfs for structure, admixture, relatedness and site-frequency-spectrum estimation. In cattle, resources such as the 1000 Bull Genomes Project are especially important because they provide high-depth reference haplotypes for imputation and variant interpretation [24]. An end-to-end lcWGS workflow integrating these steps is shown in Figure 1.

To reduce redundancy, we summarize the required QC information in Table 2 rather than repeating tool-specific thresholds in the text. At minimum, lcWGS studies should report sequencing depth and breadth, variant-level filters, imputation accuracy by MAF and population, population-structure checks, downstream sensitivity analyses and reproducibility metadata (Table 3). Thresholds should be justified according to study purpose rather than copied across species or coverage levels.

### Genotype Likelihoods, Imputation and Uncertainty-Aware Inference

The defining feature of lcWGS is genotype uncertainty. At low coverage, many heterozygous loci are supported by only one allele or by no reads. Hard genotype calls can therefore create systematic bias, particularly for heterozygosity, inbreeding, allele-frequency spectra and rare-variant analyses. For individual i at locus s, let R_is denote the observed reads and G_is in {0,1,2} denote the number of alternative alleles. A genotype likelihood is L_is(g) = P(R_is|G_is = g), which combines base quality, mapping quality and sequencing-error probabilities across reads. In a biallelic diploid model, these likelihoods are then combined with prior allele-frequency or haplotype information to obtain posterior genotype probabilities P(G_is = g|R_is, H), where H denotes reference or cohort haplotypes.

The imputed dosage used in downstream analyses is E(G_is|R_is, H) = P(G_is = 1|R_is, H) + 2P(G_is = 2|R_is, H). This dosage retains uncertainty: a genotype with posterior probabilities (0.05, 0.90, 0.05) and one with (0.45, 0.10, 0.45) can have similar hard calls or dosages under some filters but very different confidence. Therefore, reporting posterior-probability distributions and MAF-stratified dosage correlations is more informative than reporting only hard-call concordance.

Reference-panel imputation can be viewed as a hidden Markov model in which the unobserved state is the pair of reference haplotypes copied by the target animal along the chromosome [4,6,18,19]. Transition probabilities reflect recombination, and emission probabilities are determined by genotype likelihoods or observed genotypes. This formulation clarifies why imputation improves with closer reference haplotypes [9,10], stronger linkage disequilibrium, larger training sets and higher depth [13,14,15], but deteriorates for rare alleles, admixed animals and reference-mismatched indigenous breeds.

Imputation performance depends on coverage, sample size, linkage disequilibrium, allele frequency, phasing quality, relatedness and the match between target animals and reference panels [13,14,15]. Large, closely related reference panels are particularly valuable for livestock breeding populations, where pedigree structure and long haplotypes can be exploited [9,24]. However, reference panels constructed primarily from commercial breeds may perform poorly for local or indigenous populations [13]. For this reason, reference-panel design should be treated as a biological sampling problem as well as a computational problem.

Benchmarking should be performed by masking high-confidence genotypes from high-depth or SNP-array data and re-imputing them under realistic depth scenarios [6,7,8]. Accuracy should be reported by minor allele frequency, genomic region, chromosome, breed or line, and downstream endpoint. A high average genotype concordance can mask poor performance for rare variants or locally important alleles; dosage correlation, non-reference sensitivity and calibration of posterior probabilities are therefore more informative than simple concordance alone [13,14,15]. In association testing, a simple dosage-aware mixed model can be written as y = Xb + d beta + u + e, where d is the imputed dosage, u captures polygenic relatedness and e is residual error. Software such as GEMMA, GCTA-fastGWA, SAIGE and regenie can use dosage or probabilistic inputs in different ways, but they do not automatically solve low-coverage uncertainty; locus-level imputation quality and sensitivity analyses remain necessary.

## 3. Applications in Livestock and Poultry

**Figure 2:**
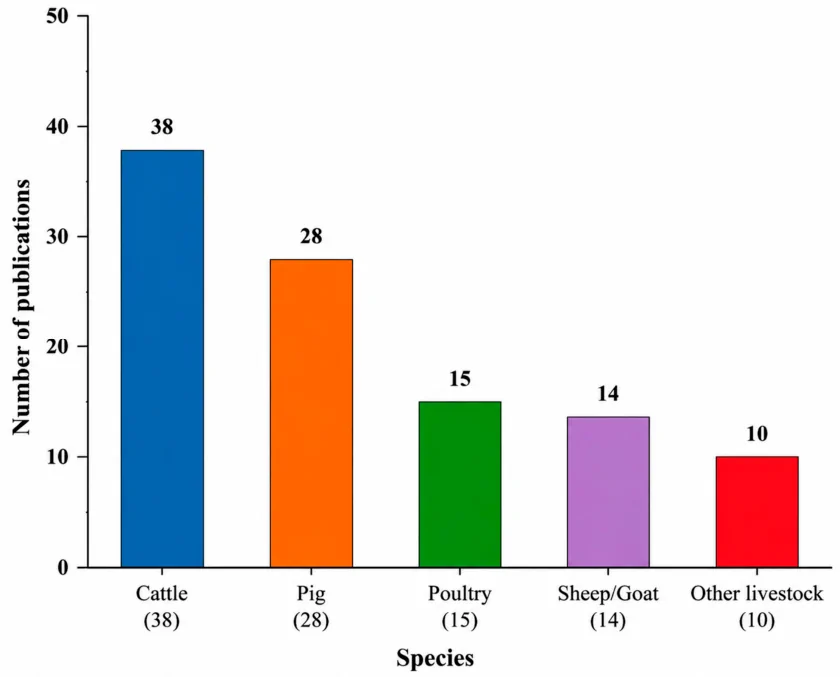
Distribution of original lcWGS studies by animal species in the targeted PubMed-based literature survey used for this review.

**Figure 3:**
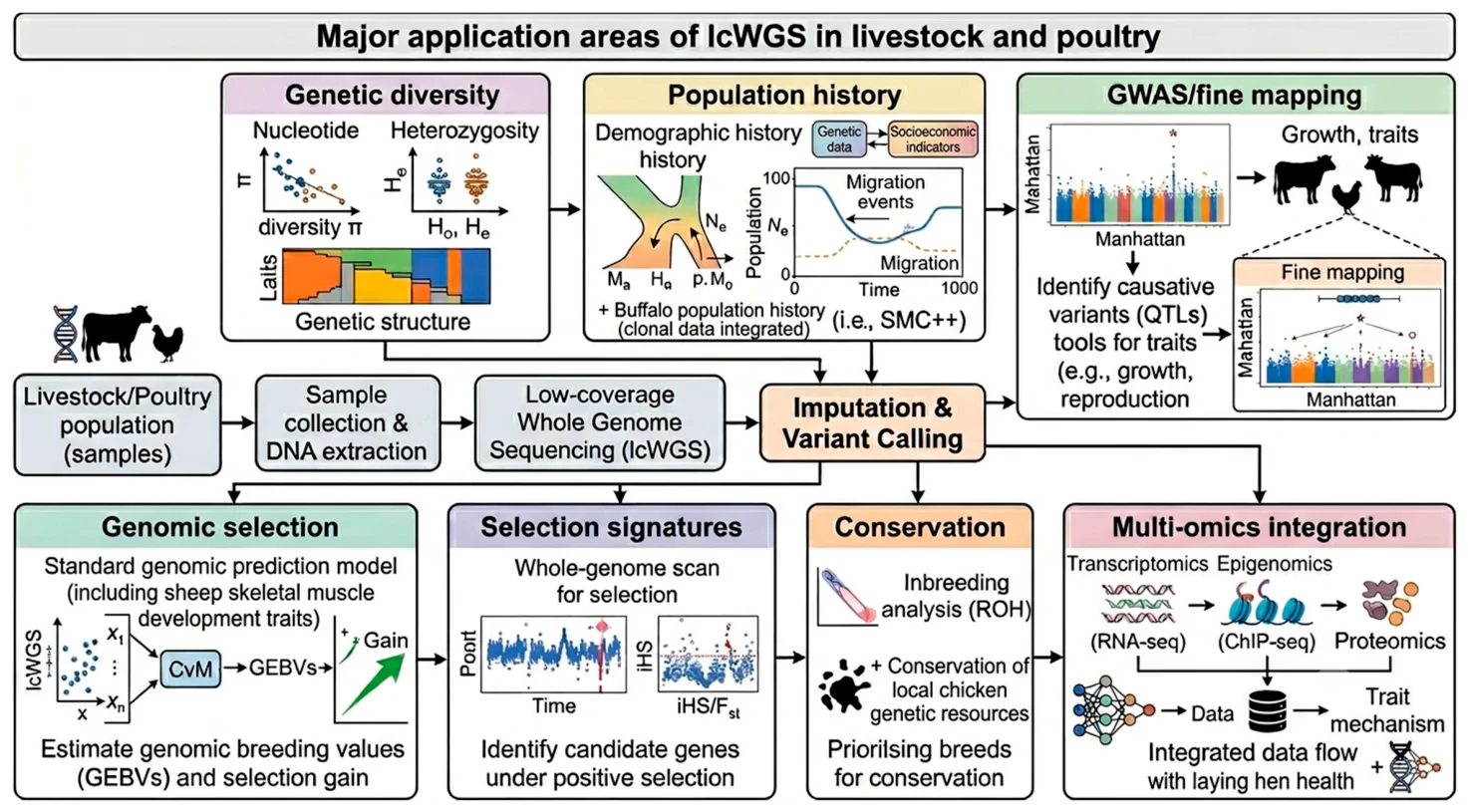
Major application areas of lcWGS in livestock and poultry.

lcWGS has four broad advantages for animal genomics: it reduces array ascertainment bias, supports large cohorts, can be reanalyzed as references improve and provides a common genomic substrate for phenotypes, pedigree, environment and multi-omics. The strength of evidence across published studies is uneven. Pig and sheep studies with close reference panels generally show high imputation accuracy and useful GWAS or genomic-prediction performance; poultry studies show clear potential but require more cross-line and cross-generation validation; cattle implementation depends strongly on the availability of high-depth reference resources; and goats remain underrepresented in lcWGS-specific studies, so evidence from high-depth goat resequencing should be treated as complementary rather than directly interchangeable. To visualize the current distribution of species-specific evidence, we conducted a targeted PubMed-based survey of original lcWGS studies included in this review (Figure 2). These major application areas are summarized in Figure 3, and representative studies are compared in Table 4.

### 3.1. Genetic Diversity and Inbreeding Assessment

Genetic diversity is the foundation of long-term breeding response, adaptation and conservation value. lcWGS can estimate nucleotide diversity, observed and expected heterozygosity, allele-frequency spectra, linkage disequilibrium decay, runs of homozygosity (ROH), genomic inbreeding and effective population size [1,12,16]. Its advantage over SNP arrays is strongest in indigenous or under-characterized breeds, where fixed arrays can miss private alleles and distort allele-frequency spectra [31,42,47]. In contrast, SNP arrays may remain sufficient for routine within-line inbreeding monitoring when marker density is adequate and array ascertainment matches the population [31,48].

For low-depth data, diversity metrics should be estimated using genotype likelihoods or posterior dosages where possible. Hard calls can underestimate heterozygosity and inflate homozygosity, especially at ultra-low depth. ROH analyses are particularly sensitive to genotype error and missing data; therefore, minimum marker density, gap length, heterozygote allowance and imputation confidence thresholds should be explicitly reported. A critical point is that increasing sample size can stabilize population-level diversity estimates, but it cannot fully recover rare alleles or long ROH segments if coverage and imputation quality are too low.

### 3.2. Population Structure, Admixture and Demographic History

lcWGS is well suited for reconstructing population structure because ancestry differences are distributed across the genome and can be inferred from large numbers of markers [1,9]. Principal component analysis, admixture modelling, phylogenetic reconstruction, identity-by-descent mapping and demographic modelling can reveal breed origins, founder effects, introgression, recent crossbreeding and genetic erosion [42,49]. The added value over arrays is largest when populations are divergent from the array discovery breeds, whereas the advantage is smaller for commercial lines already represented on high-density arrays [31,50].

The main risk is overinterpreting fine-scale structure when sequencing depth, sample size or reference-panel representation is inadequate. Studies should test cluster stability under different filters, compare genotype-likelihood-based and imputed-genotype approaches, and account for ascertainment bias when combining lcWGS with SNP-array or high-depth datasets. For poultry, where commercial lines and local breeds may have undergone strong recent selection and introgression, admixture analyses should be interpreted together with breeding history and geographic metadata rather than as purely statistical clusters.

### 3.3. Genome-Wide Association Studies and Fine Mapping

Low-coverage whole-genome resequencing can improve GWAS in livestock and poultry by increasing marker density and reducing the ascertainment bias of fixed SNP arrays. This advantage is most evident when causal or closely linked variants are poorly represented on commercial arrays, when local or indigenous populations carry population-specific haplotypes, and when a sufficiently large phenotyped cohort can be imputed using a closely matched reference panel [13,14,15]. Studies in pigs [9,10], rabbits [20], sheep [27,45] and poultry [26,51] suggest that lcWGS-derived variants can support association mapping for growth, reproduction, wool, immune-response and egg-production traits. However, lcWGS should not be assumed to automatically improve fine mapping or causal-variant discovery. If the reference panel is genetically distant, the phenotype is noisy, or the causal variant is rare, structural or poorly imputed, additional sequence markers may increase the multiple-testing and interpretation burden without narrowing the causal interval.

For lcWGS-based GWAS, genotype uncertainty should be considered in both variant filtering and association testing. In practice, imputed allele dosages or posterior genotype probabilities are preferable to hard-called genotypes because they retain more information from the imputation process. Commonly used GWAS tools, including GEMMA, GCTA-fastGWA, SAIGE and regenie, can use dosage or probabilistic genotype inputs to different extents depending on their input formats and implementation. However, in most applications, these values are treated as fixed predictors rather than as full representations of posterior genotype uncertainty from low-coverage sequencing. Therefore, studies should explicitly report whether genotype likelihoods, posterior genotype probabilities, imputed dosages or best-guess genotypes were used, and should provide locus-level imputation quality for associated variants. Candidate variants should be prioritized using multiple lines of evidence, including allele frequency, local imputation accuracy, linkage disequilibrium, biological plausibility, functional annotation and replication in independent populations. Integration with eQTLs [52,53,54], chromatin accessibility, DNA methylation, conserved regulatory elements and tissue-specific expression resources [55,56] can further improve the interpretation of lcWGS-based association signals.

### 3.4. Genomic Selection and Breeding Applications

Genomic selection predicts genomic estimated breeding values from genome-wide marker information and has transformed animal breeding [30,32]. lcWGS offers a route to sequence-level genomic selection by increasing marker density at lower per-animal cost than high-depth sequencing [33,41]. Compared with SNP arrays, lcWGS may better tag breed-specific and array-missing variants, but this benefit is conditional. It is most likely when the training population is large, closely related to selection candidates, well phenotyped and supported by a matched reference panel.

The empirical benefit of lcWGS for genomic prediction depends on trait architecture, training population size, imputation accuracy and the relationship between training and selection candidates. Studies in pigs [10,57], ducks [26], sheep [27,28] and donkeys [29] indicate that imputed low-coverage data can be used for genomic prediction and may improve accuracy relative to pedigree-only or lower-density marker approaches. Nevertheless, the most cost-effective design is not universal. In many routine commercial settings, a low- or medium-density SNP array plus imputation to a strong reference panel may still be preferable because it is standardized, inexpensive and operationally simple. Breeding programs should compare array genotyping, lcWGS at several depths and targeted sequencing under the same validation framework before routine implementation.

### 3.5. Selection Signatures and Environmental Adaptation

Domestication, breed formation, artificial selection and local environmental pressures leave genomic footprints that can be detected through allele-frequency differentiation, haplotype homozygosity, nucleotide-diversity reduction and ROH enrichment [58,59,60]. Common statistics include FST, nucleotide-diversity ratios, Tajima’s D, iHS, XP-EHH, ROH-based metrics and composite likelihood methods. lcWGS enables these scans to be performed in larger and more diverse cohorts, but the benefit depends on whether the statistic can tolerate genotype uncertainty [1,11,16]. Frequency-based statistics can often be estimated from likelihoods or dosages [11,16], whereas haplotype-based tests usually require more reliable phasing and imputation [1,9].

Selection-scan studies in cattle, pigs, sheep, goats and chickens have identified candidate regions associated with growth, reproduction, meat quality, immune function, heat tolerance, hypoxia response and other adaptive traits. For example, high-altitude adaptation studies across domestic mammals have highlighted convergent signals in hypoxia-related pathways, while chicken studies from tropical and temperate regions have detected candidate genes related to heat tolerance and climatic adaptation [58,59]. In sheep and goats, combining population genomic statistics with environmental variables can reveal genes involved in thermal, arid and high-altitude adaptation [46,60].

Because demographic events can mimic selection, single-statistic scans should be avoided. Stronger designs combine multiple statistics, environmental association tests, phenotype association, cross-population replication and functional evidence. Candidate regions identified from lcWGS should be prioritized using gene expression, enhancer annotations, eQTL/sQTL resources and pathway-level analyses rather than relying solely on outlier windows. High-depth or long-read validation is particularly important when the candidate mechanism may involve copy-number variation, inversions or other structural variants.

### 3.6. Conservation Genetics and Genetic Resource Management

lcWGS has considerable value for conservation genetics because many local breeds have small population sizes, incomplete pedigrees and limited array resources. Genome-wide data can identify genetically unique populations, quantify loss of diversity, monitor introgression from commercial lines, estimate effective population size and inform mating or cryopreservation decisions [47,48,50]. For poultry, whole-genome studies of indigenous chickens show that local breeds can retain unique genetic structure and adaptive variation, while also revealing variable introgression from commercial lines [42,49]. For goats and other underrepresented species, current high-depth studies demonstrate the biological importance of sequencing local breeds [46], but dedicated lcWGS benchmarking is still needed before routine conservation use.

In practice, lcWGS can help define conservation units, select founders for gene banks, minimize redundancy among cryopreserved individuals and design mating plans that reduce future inbreeding. The approach is most powerful when combined with phenotypic characterization, geographic origin, production environment and cultural or historical information. A purely genomic ranking of breeds may overlook traits that are locally important but poorly measured; therefore, conservation decisions should integrate genomic, phenotypic and socioeconomic evidence. Data governance is also part of conservation: communities, farms and regions maintaining local genetic resources should be recognized as stakeholders, and benefit sharing, consent, metadata control and controlled access should be planned before data release.

## 4. Technical Challenges and Reporting Standards

Despite its advantages, lcWGS remains vulnerable to several sources of bias. Rather than repeating each limitation in multiple sections, Table 5 consolidates the major risks and mitigation strategies. The most important message is that low depth, imputation mismatch, reference bias, population stratification, rare variants, structural variants, computational reproducibility and data governance interact with each other [13,14,15]. A weak reference panel can reduce rare-allele accuracy [13,14,15], distort GWAS signals [9,10] and worsen portability across breeds even when average imputation concordance appears high.

Reference bias remains a central challenge. Reads carrying non-reference alleles may align less efficiently to a single linear reference genome, particularly in divergent breeds or species with incomplete reference representation. This can distort allele-frequency estimates, population differentiation and selection scans. Multiple-reference methods [37], breed-specific assemblies [35,36], improved reference genomes and graph pan-genomes [38,39] can reduce this bias but require additional computational infrastructure.

Structural variation is another area where lcWGS should not be oversold [35,36]. Short-read lcWGS is effective for SNP and small-indel inference after imputation, but it is less reliable for copy-number variants, mobile element insertions, inversions, complex rearrangements and reference-missing sequence. Long-read assemblies [36] and deep-sequenced representative panels [35] are therefore needed to build SV catalogs and pan-genome resources [38,39] that can be projected onto larger lcWGS cohorts.

Transparent reporting is essential. Studies should describe sample selection, sequencing design, reference genome, read-processing pipeline, variant filters, imputation software, reference-panel composition, validation design and downstream sensitivity analyses. Data sharing should follow FAIR principles [44] while respecting commercial confidentiality, national regulations and the rights and interests of communities maintaining local genetic resources [47,48]. When raw genotypes cannot be public, authors should provide controlled-access routes, summary statistics, reference-panel descriptions, metadata dictionaries and code sufficient for reproducibility [44].

## 5. Future Perspectives: From Low-Cost Genotyping to Functional Interpretation

**Figure 4:**
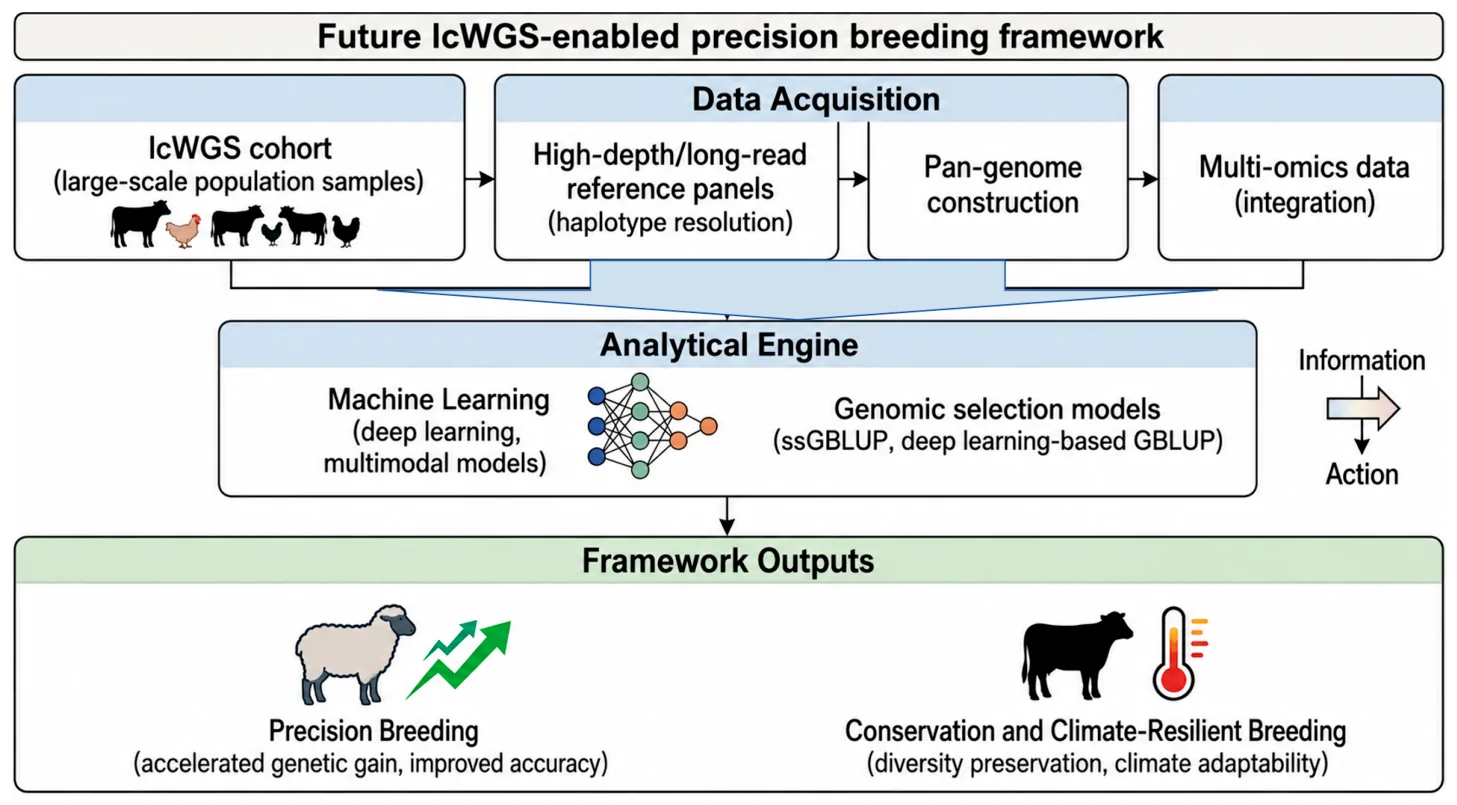
Future lcWGS-enabled precision breeding framework.

The next stage of lcWGS in livestock and poultry will be defined less by sequencing alone than by implementation. Population-scale lcWGS can provide broad, cost-effective sampling, while high-depth short-read sequencing, long-read sequencing and chromosome-scale assemblies provide the reference resources needed to interpret complex variation. Graph pan-genomes are promising because they represent multiple haplotypes and non-reference sequences, reducing the limitations of a single linear reference [36,37,38,39]. However, several bottlenecks still prevent wider routine use: insufficient breed-specific reference panels, uncertain performance for rare and structural variants, inconsistent software metrics, limited cross-generation validation, storage and computation costs, lack of standardized reporting, and governance restrictions on commercial or local-breed data. An integrated future precision-breeding framework enabled by lcWGS is presented in Figure 4.

Functional annotation resources will increasingly determine whether lcWGS findings can move from statistical association to biological mechanism. FAANG and FarmGTEx have generated or coordinated epigenomic, transcriptomic and regulatory-variant resources for major farm animal species [55,56,61,62]. CattleGTEx [52], PigGTEx [53] and ChickenGTEx [54] demonstrate how multi-tissue expression and splicing data can connect variants with regulatory mechanisms and economically important traits. These resources can be used to prioritize candidate variants from lcWGS GWAS [63,64], interpret selection signatures and improve genomic prediction.

Multi-omics integration is especially important for complex traits such as fertility, feed efficiency, immune response, heat tolerance and egg production. A future lcWGS study should ideally combine genotype dosages with phenotype records, longitudinal environment, gene expression, chromatin accessibility, methylation, proteomics or metabolomics. Such integration can identify not only which loci are associated with traits, but also in which tissues, developmental stages and environmental contexts those loci act.

Machine learning and artificial intelligence can further enhance lcWGS by improving imputation, feature selection, genomic prediction and causal-variant prioritization [65,66]. However, these methods should be evaluated against simpler GBLUP, Bayesian and mixed-model baselines, externally validated across breeds or generations, and interpreted at the level of variants, genes, pathways or regulatory elements. Black-box accuracy without biological interpretation or operational validation is not sufficient for routine breeding decisions.

In summary, the incremental contribution of this review is to place lcWGS in the specific decision environment of livestock and poultry rather than to treat it as a generic sequencing technology. We highlight how family structure, inbred or line-specific populations, breeding reference panels, indigenous breeds, poultry lines, conservation goals and commercial data restrictions change the balance among sample size, coverage, imputation and downstream utility. This framing complements earlier general introductions to lcWGS and is intended to help breeders, conservation geneticists and animal genomics researchers choose when lcWGS is the right tool and when it is not.

## 6. Conclusions

Low-coverage whole-genome resequencing is advancing livestock and poultry genomics from marker-limited genotyping toward population-scale sequence inference. Its principal value lies in enabling broad and representative sampling while retaining genome-wide information through genotype likelihoods, haplotype reconstruction and imputation. Consequently, the performance of lcWGS is determined not by sequencing depth alone, but by the coordinated design of cohort size, phenotype collection, reference-panel composition, population structure, imputation strategy and downstream statistical analysis. When these elements are appropriately aligned, lcWGS provides a versatile genomic framework for investigating population diversity, identifying trait-associated loci, improving genomic prediction, characterizing adaptive variation and supporting the management of animal genetic resources.

The continued development of lcWGS will increasingly depend on its integration with complementary genomic and functional resources. High-depth sequencing of representative animals, long-read assemblies and graph pan-genomes can strengthen reference panels and improve the representation of breed-specific haplotypes and structural variation. Functional genomic resources, including FAANG and FarmGTEx, together with environmental and phenotypic data, can further connect sequence variation with regulatory mechanisms and complex traits. Within such integrated frameworks, interpretable statistical and machine-learning models may facilitate the transition from variant discovery to biological explanation and breeding application. Overall, lcWGS should be regarded as a scalable population-genomic strategy rather than merely a lower-cost genotyping method. Its thoughtful implementation has the potential to accelerate genetic improvement, strengthen climate-resilient breeding and promote the sustainable conservation and utilization of livestock and poultry genetic diversity.

## Figures and Tables

**Table 1:** Practical decision framework for choosing lcWGS, SNP arrays, GBS or high-depth WGS.

Primary Objective	lcWGS	Another Strategy	Critical Validation
Routine genomic evaluation within a well-characterized commercial line	The breeding program wants sequence-level marker density, breed-specific variants or a unified platform across lines.	A validated low-cost SNP array already captures the relevant haplotypes and turnaround time is the dominant constraint.	Compare prediction accuracy, bias and cost against the current array in the same validation population.
GWAS and fine mapping	Causal or tagging variants may be absent from arrays, and a large phenotyped cohort is available.	The trait is rare, sample size is small, or associated variants are expected to be very rare or structural.	Report locus-level imputation Rsq/DR2, MAF-stratified accuracy and replication or functional evidence.
Indigenous breed diversity and conservation	Array ascertainment bias is expected and private/local alleles are important.	The population is extremely small and the main aim is complete cataloguing of rare or structural variants.	Use genotype-likelihood or dosage-aware metrics and include high-depth representatives when possible.
Reference-panel construction, SV discovery or diagnostic use	lcWGS is used only for broad cohort projection after reference animals are sequenced deeply.	Per-individual genotype certainty, rare variants or complex SVs are the direct targets.	Use high-depth short-read, long-read or targeted validation for key animals and loci.

**Table 2:** Comparison of lcWGS, SNP arrays, genotyping-by-sequencing and high-depth WGS for livestock and poultry genomics.

Strategy	Typical Marker Spectrum	Strengths	Limitations	Best-Use Scenarios	Recommended Quality Metrics
Low-coverage whole-genome sequencing (lcWGS; usually ~0.1–5×)	Genome-wide SNPs and small indels discovered from sequencing reads and refined by genotype likelihoods or imputation; can capture population-specific and some low-frequency variants, but structural variants are usually incompletely resolved without high-depth or long-read support [1,4,9,20,25].	High marker density; lower ascertainment bias than fixed arrays; scalable for large cohorts; flexible across breeds and species; suitable for imputation-aware GWAS, genomic selection, diversity analysis and selection scans [1,9,26,27,28].	Genotype uncertainty, allele dropout and missingness at low depth; imputation accuracy depends on coverage, sample size, linkage disequilibrium, relatedness and reference-panel match; rare-variant and SV accuracy remain weaker than high-depth WGS [1,13,14,15].	Large-scale GWAS, genomic selection, genetic diversity monitoring, population structure, selection signatures and conservation surveys where the goal is to maximize population-level information under a fixed budget [9,25,26,29].	Mean and variance of depth; breadth and uniformity of coverage; duplicate rate; sample/variant missingness; genotype likelihood calibration; imputation Rsq/DR2; dosage/high-depth or array concordance; MAF-stratified accuracy; Ti/Tv ratio; relatedness and population-stratification checks [4,13,16,17].
SNP arrays	Pre-selected biallelic SNPs at low-, medium- or high-density panels; enriched for common variants present in the discovery breeds; limited detection of novel, rare, breed-specific variants, indels and structural variants [13,30,31].	Standardized, inexpensive per sample in established species; high genotype call rates; simple quality control; mature downstream pipelines; widely used for routine genomic evaluation, parentage testing and within-breed selection [30,32,33].	Fixed marker content; ascertainment bias toward discovery populations and common alleles; reduced informativeness in divergent or indigenous breeds; limited fine mapping if causal variants are absent or poorly tagged [13,31,33].	Routine breeding programs in well-characterized commercial populations; genomic relationship estimation; parentage verification; rapid deployment when validated species- or breed-specific arrays are available [30,32].	Sample and SNP call rate; cluster separation; GenCall/quality score; Hardy-Weinberg equilibrium; MAF distribution; Mendelian errors; duplicate concordance; sex checks; heterozygosity outliers; genomic relationship consistency [30,31,33].
Genotyping-by-sequencing (GBS)/reduced-representation sequencing	SNPs located near restriction-enzyme cut sites or targeted regions; marker number and distribution depend on enzyme choice, library design and read depth; fewer genome-wide markers than WGS and more missing data than arrays [33,34].	Lower cost than high-depth WGS; supports de novo marker discovery; useful when arrays are unavailable or poorly optimized; adaptable to non-model breeds and species; can be used for population genetics and genomic prediction with adequate sample size and imputation [34].	Uneven genomic coverage; high missingness; allele dropout; strong dependence on DNA quality, digestion efficiency, multiplexing and bioinformatic filters; lower reproducibility across protocols or batches than fixed arrays [34].	Exploratory population genomics, early-stage marker discovery, non-model livestock or aquaculture species, and low-cost genomic selection when a reduced-representation marker set is acceptable [33].	Per-sample read count; number of retained loci; locus depth distribution; missingness before and after imputation; duplicate concordance; heterozygosity excess; enzyme/insert-size reproducibility; imputation accuracy; batch effects [34].
High-depth whole-genome sequencing (high-depth WGS; commonly ≥10–30×)	Comprehensive genome-wide SNPs, indels and rare/private variants; improved detection of CNVs and structural variants with appropriate callers; strongest resolution when combined with long-read sequencing or pan-genome references [11,35,36].	Highest per-sample genotype confidence; enables direct variant discovery, rare causal-variant detection, SV cataloguing, reference-panel construction and validation of lcWGS imputation results [11,35].	High sequencing, storage and computational cost; smaller sample sizes under fixed budgets; reference bias and difficult regions still remain; functional interpretation of rare variants and SVs requires additional evidence [35,36,37].	Sequencing key founders, sires/dams or reference animals; building high-quality reference panels; discovering rare Mendelian or large-effect variants; validating GWAS signals; constructing pan-genome/SV resources [35,36,38,39].	Mean depth and callable genome; coverage breadth and uniformity; mapping and base quality; duplicate rate; contamination; Ti/Tv ratio; heterozygous/homozygous ratio; Mendelian consistency; variant-quality recalibration; SV caller concordance or orthogonal validation [11,17,35,36].

**Table 3:** Recommended QC and benchmarking metrics for lcWGS studies.

QC Domain	Core Metrics	Recommended Benchmarking/Thresholds	Why It Matters	Reporting Items and Supporting References
Sequencing QC	Total reads; Q20/Q30; per-base quality; adapter content; GC distribution; duplication rate; mean depth; coverage breadth; depth variance; batch balance.	Predefine minimum reads/depth per sample; inspect outliers in depth, GC and duplicate rate; remove or resequence samples with extreme coverage or contamination; randomize biological groups across lanes/batches.	Low-pass data are sensitive to uneven coverage, library effects and missingness. Sequencing QC prevents batch effects from being converted into false genotype or allele-frequency differences.	Report sequencing platform, read length, library protocol, target depth, actual depth distribution, coverage breadth and sample exclusions [7,35,36].
Variant QC	Mapping quality; base quality; genotype likelihood distribution; allele balance; site depth; missingness; MAF; Hardy-Weinberg equilibrium; Ti/Tv ratio; repeat/low-complexity annotation.	Prefer genotype-likelihood or imputation-aware filters before hard calls; remove low-mappability, extreme-depth and high-missingness sites; apply MAF- and depth-stratified QC rather than one universal cutoff.	Variant-level errors can inflate false-positive GWAS signals, distort FST/selection statistics and reduce imputation accuracy, especially in divergent breeds or repetitive regions.	Report reference genome version, aligner, variant caller, site filters, callable genome definition and variant counts before/after filtering [11,13,35,36].
Imputation QC	Phasing error proxies; imputation Rsq/DR2; posterior genotype probability; dosage concordance; non-reference discordance; switch error when trios or truth data exist; MAF-stratified accuracy.	Benchmark against high-depth WGS, SNP arrays, trios, replicated samples or masked genotypes; evaluate accuracy by MAF class, breed/population, chromosome and sequencing depth.	Imputation is the central error-control step in lcWGS. Average accuracy can hide poor performance for rare alleles, divergent breeds or weakly represented haplotypes.	Report imputation software/version, reference-panel size and ancestry, phasing strategy, validation design, Rsq/DR2 distributions and MAF-stratified concordance [4,13,14,15].
GWAS/GS QC	Phenotype QC; covariates; relatedness matrix; principal components; genomic inflation; effective sample size; cross-validation; prediction accuracy; calibration and bias.	Use dosage- or uncertainty-aware models where possible; control population structure and relatedness; separate training/validation groups by family, generation or breed when testing portability.	Residual structure, relatedness and imputation uncertainty can bias association statistics or overestimate genomic prediction performance.	Report phenotype edits, model formula, covariates, kinship method, multiple-testing procedure, validation scheme, accuracy metric and sensitivity analyses [27,40,41].
Population-genetic QC	PCA/ADMIXTURE stability; pairwise relatedness; heterozygosity; inbreeding/ROH; LD decay; Ne; FST; nucleotide diversity; Tajima’s D; allele-frequency uncertainty.	Use genotype-likelihood or dosage-aware methods for low-depth data; repeat analyses under alternative filters; remove close relatives when required; validate fine-scale structure with independent data if possible.	Population-genetic statistics can be biased by low depth, sample imbalance, demography, reference bias and admixture; sensitivity analysis is essential.	Report sample origin, breed/population definitions, missingness/depth filters, method settings, number of SNPs used and robustness checks [16,21,22,23,42].
Reproducibility reporting	Workflow provenance; software versions; parameters; reference-panel access; metadata completeness; random seeds; container or environment files; data availability.	Use scripted or containerized workflows; archive key parameters and QC thresholds; share summary statistics, filtered variant metadata and reference-panel descriptions when raw data cannot be public.	Transparent reporting allows other groups to benchmark lcWGS designs, reproduce genotype-processing decisions and compare results across breeds, species and laboratories.	Report pipeline diagram, software versions, reference genome accession, filtering code, metadata schema, FAIR-compliant data or controlled-access statements [43,44].

Note: Thresholds should be justified according to sequencing depth, species, population structure, reference-panel composition and downstream analysis goals. Rsq/DR2 values are software-specific and should not be compared across tools unless the same validation procedure is used.

**Table 4:** Comparative interpretation of representative sequencing applications relevant to lcWGS implementation in livestock and poultry.

Species/Population	Cohort and Coverage	Reference/Imputation Strategy	Application	Accuracy or Validation Evidence	Main Practical Outcome	Critical Interpretation
Duroc pig	2869 boars; 0.73×	BaseVar-STITCH	21-trait GWAS	Millions of imputed SNPs; locus-level validation reported [9]	High-resolution QTL mapping beyond fixed arrays	Large related cohort and within-breed LD made lcWGS effective; rare variants and SVs remained weakly resolved.
Large White pig	1423 pigs plus 30 key progenitors; lcWGS	Ref_LG/Mix_HLG/Within_LG	Reproductive GP and GWAS	Ref_LG accuracy about 0.9899 [10]	18 candidate genes and strong genomic prediction utility	Performance depended on matched progenitors/reference design; transfer to divergent breeds should not be assumed.
Hu sheep	3007 lambs; 1.24×	Internal Hu reference panel	Birth-weight GWAS	Large within-breed sample supported fine mapping [45]	QTLs on chromosomes 6 and 9; PLAG1-linked signal and FecB effect	Useful model for local-breed studies with internal reference support; cross-breed portability remains untested.
Sheep body weight/milk yield	Breed-specific cohort; lcWGS	Similar-background reference panels	GWAS and genomic selection	Imputation and prediction evaluated within similar genetic backgrounds [27]	Supported lcWGS-based genomic evaluation in sheep	Shows that reference-panel match is more important than average depth alone.
Muscovy duck	975 sequenced of 1491 phenotyped; about 0.84×	GBLUP/SSGBLUP plus LD/kernel models	Egg-production genomic selection	Prediction improved 12.3–43.9% over PBLUP [26]	Improved breeding-value prediction from low-pass sequence data	Promising poultry evidence, but cross-line and later-generation validation are still needed.
Angora rabbit	627 low-pass animals; 3.84× mean	BaseVar-STITCH and Beagle pipelines	Wool-trait GWAS	QTLs explained 0.4–7.5% [20]	FGF10 highlighted as a candidate gene	Higher low-pass depth increased confidence but reduced cost advantage relative to 0.5–1× designs.
Dezhou donkey	617 donkeys; simulated 1× and above	BaseVar-STITCH	Genomic prediction and imputation design	Accuracy exceeded 0.95 at 1× with at least 400 sequenced animals [29]	Provides a design guide for species with limited genomic resources	Simulation and cohort composition should be validated before routine deployment.
Cattle	Species-level lcWGS evidence and reference-panel resources	1000 Bull Genomes and other high-depth panels	Prospective lcWGS implementation	High-depth reference resources improve imputation potential [24,25]	Supports transition from arrays to sequence-based genotyping in cattle	Implementation depends on breed representation, data access and validation in the target evaluation system.
Goats	High-depth WGS examples; lcWGS evidence still limited	High-depth resequencing rather than low-pass imputation	Diversity and selection signatures	Hainan black goat study identified 23,608,983 SNPs in 33 goats [46]	Shows the biological value of sequencing for local goats	Included as a reference benchmark; true goat lcWGS studies are still needed.

**Table 5:** Common sources of bias in lcWGS and recommended mitigation strategies.

Bias Source	How Bias Arises	Downstream Impact	Mitigation Strategy	Minimum Reporting
Low depth	Sparse coverage; allele dropout; uncertain heterozygotes.	Biased allele frequency, weak GWAS power and unstable genomic relationships.	Use likelihoods/dosages; optimize depth-sample trade-off; validate with arrays or high-depth data [1,12,16].	Target/realized depth; coverage breadth; missingness; exclusions.
Imputation mismatch	Reference panel differs in ancestry, LD or haplotype composition.	Poor rare-allele accuracy; false or missed GWAS/GS signals.	Use breed-aware panels; include key ancestors; report MAF/population-stratified Rsq/DR2 [4,13,14,15].	Reference size, ancestry, phasing method and validation design.
Reference bias	Non-reference alleles map or pass filters less efficiently.	Distorted diversity, FST, selection scans and association signals.	Use improved assemblies, pan-genomes, graph/multiple-reference approaches when available [37,38,39].	Reference version; aligner; mappability and repeat filters.
Population stratification	Breed structure, admixture, family and batch effects.	Inflated statistics and overestimated prediction accuracy.	Use PCA/ADMIXTURE/kinship; fit covariates; use within-breed or stratified validation [1,42,49].	Sampling, breed labels, covariates, kinship and validation split.
Rare variants	Low allele counts and weak imputation support.	False negatives or low-confidence rare associations.	Apply MAF-stratified QC; validate candidates using high-depth or functional evidence [4,12,45].	MAF threshold; rare-variant concordance; posterior filters.
Structural variants	Low-pass short reads poorly resolve CNVs and complex SVs.	Causal SVs missed; incomplete interpretation of GWAS/adaptation.	Integrate high-depth WGS, long reads, SV maps and pan-genomes [35,36,38].	SV caller, SV classes, validation platform and excluded regions.
Computational reproducibility	Different tools, filters, seeds and parameters alter genotype sets.	Low cross-study comparability and hidden analytical choices.	Use scripted/containerized workflows and sensitivity analyses [17,44].	Software versions, commands, thresholds, seeds and code access.
Data governance	Commercial or genetic-resource restrictions limit data sharing.	Limited reproducibility and unequal reference-panel access.	Apply FAIR principles, controlled access and data-use agreements [44,47,48].	Access route, restrictions, metadata, ethics and consent.

## Data Availability

All relevant data are included in the article. Further information can be obtained from the corresponding author upon reasonable request.
